# Current status and factors influencing research ethics knowledge and attitudes among pediatricians: a cross-sectional survey

**DOI:** 10.3389/fmed.2025.1697458

**Published:** 2026-01-15

**Authors:** Bingya Zheng, Na Zhang, Hongwen Qin, Lihui Zhu

**Affiliations:** 1 Department of Child Healthcare, NHC Key Laboratory of Birth Defect for Research and Prevention (Hunan Provincial Maternal and Child Health Care Hospital), Changsha, China; 2 Early Clinical Research Center, Hunan Cancer Hospital, The Affiliated Cancer Hospital of Xiangya School of Medicine, Central South University, Changsha, China; 3 Ethics Office, Hunan Children's Hospital, Changsha, China; 4 Department of Nursing, Hunan Cancer Hospital/The Affiliated Cancer Hospital of Xiangya School of Medicine, Central South University, Changsha, China

**Keywords:** child subjects, ethical review, influencing factors, pediatricians, research ethics

## Abstract

**Background:**

Pediatricians play a pivotal role in clinical research, and their research competence is essential for advancing hospital-based studies and fostering the development of pediatric disciplines. Given the unique vulnerabilities of the pediatric population, adherence to research ethics is of paramount importance. Therefore, it is imperative for pediatricians to actively acquire knowledge related to research ethics in order to safeguard research quality, ensure the safety of research subjects, and enhance the standardization of ethical practices in research.

**Methods:**

A cross-sectional survey was conducted among 256 pediatricians in Hunan Province, China, between October 2023 and April 2024 using a convenience sampling approach. Data were collected through a general information form and a questionnaire assessing pediatricians’ knowledge of and attitudes toward research ethics.

**Results:**

The total research ethics knowledge score for the pediatricians was 70.55, and the total attitude score was 92.87. The three lowest-scoring entries in the knowledge dimension were the basic principles of medical ethics and two situational judgment questions. The results of the multiple linear regression analysis showed that degree, technical title, whether there was an ethics committee in our hospital, and whether they participated in training related to ethical knowledge were the four factors influencing the knowledge of research ethics (*F* = 9.270, *p* < 0.001, R2 = 18.3%). The number of clinical research projects attended, the number of clinical research projects chaired, and whether or not they had participated in training related to ethical knowledge were the three factors influencing attitude toward research ethics (*F* = 4.259, *p* < 0.001, R2 = 7.80%).

**Conclusion:**

Pediatricians demonstrated a moderate level of research ethics knowledge but held largely positive attitudes. Whether or not they attended training related to ethical knowledge affected both knowledge and attitude scores. Notably, degree, technical title, and the presence of an ethics committee in our institution affected the knowledge score but not the attitude score.

## 1 Introduction

Medical scientific research is an important means of promoting medical development. As pediatricians are one of the main forces in the development of research in hospitals, mastering issues related to research ethics is a fundamental principle for protecting the rights of subjects (1). Children are a special and vulnerable group in scientific research (2), and pediatric clinical research faces more confusion and dilemmas than adult medicine across the entire spectrum of clinical medicine research. The complexity of the children’s informed consent process, the conditions of pediatric care, reimbursement compensation criteria, and the lack of understanding by ethics committees of the specificities of pediatrics (3) are characterized. With the development of the social economy, children’s health and other issues are receiving increasing attention, and scientific research projects involving children are increasing annually. Safeguarding the legitimate rights, interests, and physical and mental health of children while maintaining scientific research innovativeness has always been an important aspect of the management process of pediatric clinical research projects that cannot be ignored (4). Therefore, pediatricians are more likely to take the initiative to follow the ethical principles of medical research and to properly handle ethical issues in the research process.

Research ethics refers to the ethical norms and codes of conduct between researchers and collaborators, subjects, and the ecological environment, which tests the academic value orientation and human moral pursuit of researchers (5). In the academic context of global prosperity, the medical research community has witnessed a more serious phenomenon of incorrect academic ethos and corruption of academic morality (6). In 2017, Springer Nature announced that 107 papers published by Chinese scholars in the journal Tumor Biology had been retracted due to peer-review fraud (7). A large portion of the scholars involved were clinicians. In 2018, Associate Professor He Jiankui of the Southern University of Science and Technology announced that a pair of twin babies had been given antibodies to HIV through gene editing technology, which was listed as one of the three worst international scientific events of 2018 (8), a serious violation of scientific research ethics and legal norms. Existing surveys in some regions suggest that knowledge and compliance with pediatric research ethics standards among healthcare professionals may be suboptimal (9, 10), and ethical shortcomings contribute significantly to research retractions in pediatrics (11). However, pediatricians are the main practitioners of children’s health research. There is still a significant lack of detailed data on their capabilities in research ethics, and this situation is particularly pronounced in China. Notably, the risk of retraction due to the “formalization of ethical review” is 2.7 times higher in developing countries than in developed countries (12), highlighting the urgency of strengthening pediatric research ethics in developing countries. In recent years, countries have introduced a series of research ethics policies involving clinical research and ethical review (13–15). However, the implementation of ethical awareness in research has not penetrated the research work of pediatric researchers, and thus may still not adequately protect research subjects. Therefore, a series of ethical issues arising from research will be an important issue facing the pediatric research process in the future.

With the increase in the number and complexity of scientific research projects, pediatricians, as the main body conducting pediatric-related research, are not only the actual operators of the research but also in direct contact with the subject children (16). Their comprehensive ethical level, such as research ethics knowledge and ethical attitudes, has a direct impact on the quality of research and the safety of subjects. At this stage, there is little research on the ethical knowledge and attitudes of pediatricians in the process of conducting scientific research. In global research, there have been investigations into the research ethics knowledge and attitudes of doctoral students in universities and medical staff in tertiary hospitals (17, 18). In China, few studies on research ethics have been conducted, and the main cities where ethical research has been conducted are Beijing, Shanghai, Guangzhou, and Chongqing. These studies mostly focused on the current status of research training, training needs, and ethical review perceptions of clinical researchers, medical staff, and clinicians in a particular province or hospital (19, 20). The investigation of pediatricians’ knowledge and attitudes toward research ethics is still insufficiently focused. As key contributors to advancing hospital research and promoting high-quality pediatric disciplines, and given their work with the special population of children, pediatricians can better implement ethical behaviors only by mastering the knowledge of research ethics and correctly understanding the deeper connotations of research ethics. Therefore, this study aimed to understand the knowledge and attitudes of pediatricians toward research ethics through a cross-sectional survey, analyze the factors that affect the knowledge and attitudes of pediatricians toward research ethics, and provide evidence-based recommendations for the development of a systematic education and training program for pediatricians on research ethics.

## 2 Methods

### 2.1 Study design

A descriptive cross-sectional study was conducted in Hunan Province, China, using an online survey.

### 2.2 Participants

Participants were recruited from registered pediatricians in Hunan Province, China. A convenience sampling method was adopted. The sampling frame consisted of pediatric departments within tertiary hospitals across the province, accessed through the professional networks of the research team and collaborating hospital contacts. The inclusion criteria were registered pediatricians, voluntary participation in this study, and native Chinese speakers. Ultimately, the participating pediatricians were mainly from 10 cities in the province, including Zhuzhou, Chenzhou, Changde, and Zhangjiajie. While the sample is not statistically representative, its size and diversity are suitable for the exploratory aims of the study.

### 2.3 Measures

#### 2.3.1 Demographics and professional experiences

The general information of the participants included gender, age, degree, hospital rank, technical title, years of practice, number of clinical research projects participated in, number of clinical research projects chaired, whether there was an ethical committee in their hospital, and whether they had participated in training related to ethical knowledge.

#### 2.3.2 Research ethics knowledge, attitude questionnaire

Questionnaire Development: First, an initial item pool was generated based on a comprehensive review of established research ethics assessment tools and relevant national guidelines (21–23). Second, these items were discussed and refined in a series of research team meetings involving clinical professionals (pediatricians) and research methodologies to ensure relevance and clarity. Content validity was assessed by having five experts (two in pediatric clinical research, two in research ethics, and one in survey methodology) rate each item for relevance and clarity. Each expert independently rated the relevance and clarity of each item on a 4-point Likert scale (1 = not relevant/clear, 4 = highly relevant/clear). The Item-level Content Validity Index (I-CVI) was calculated as the proportion of experts giving a rating of 3 or 4. All retained items had an I-CVI of 1.0, and the Scale-level Content Validity Index/Average (S-CVI/Ave) for the entire questionnaire was 0.96.

Pilot Testing and Reliability measurement: The revised questionnaire was piloted among a convenient sample of 20 pediatricians in Hunan Province, who were not included in the main study. The pilot aimed to test the comprehensibility of items, approximate completion time, and identify any ambiguous wording. Based on feedback, minor adjustments were made to the phrasing of several items. In the main study sample (*n* = 256), the internal consistency reliability of the questionnaire was good, with a total Cronbach’s alpha coefficient of 0.835. The Cronbach’s alpha for the knowledge and attitude subscales were 0.922 and 0.812, respectively, indicating acceptable to excellent reliability.

Questionnaire determination: The knowledge dimension included ethical concepts, the basic principles of medical ethics, the structure of ethical committees, knowledge of Good Clinical Practice in Clinical Trials, and cases of ethical problems in clinical research projects. The research ethics knowledge questionnaire was scored out of 100 points. Questions 1–8 were worth 7 points each, questions 9–11 were worth 10 points each, and questions 12–13 were situational judgment questions, worth 7 points each. Higher scores indicated better knowledge of research ethics. The attitude dimension included attitudes toward the importance of ethical review and the need for a Good Clinical Practice (GCP) certificate for clinical research. Each item was scored on a 5-point Likert scale, with “strongly disagree, relatively disagree, not sure, relatively agree, strongly agree” scored from 1 to 5; higher scores indicated more positive research ethics attitudes among the study participants. The total scores for knowledge and attitude were calculated using standardized scores, calculated as: mean score/total score×100% (24). The full questionnaire items are provided as Supplementary material 1.

### 2.4 Procedure

The survey for this study was conducted using an online questionnaire. Today, almost every physician working in a hospital has access to various social media and Internet platforms. This online survey was initiated using the electronic data collection tool “Questionnaire Star”.^1^ The link to the questionnaire was posted on the Internet and sent to different hospitals in various cities in Hunan Province by members of the research team or hospital contacts. These hospitals were invited to share the survey link with their pediatricians. It was completely voluntary whether or not to fill out the survey. The survey link was available for 1 week (March 5, 2024 March 12, 2024). Each IP address and account could only respond once to avoid duplication of responses. A total of 280 pediatricians completed the survey questionnaire. Based on the total number of items in this survey, it was estimated that completing all items would take approximately 10 min. To ensure the quality of the data, we eliminated questionnaires that were completed in a very short period of time (<3 min) or a considerable amount of time (about 20 min), leaving 256 valid questionnaires.

### 2.5 Data analysis

All data were analyzed using SPSS version 26.0. Data for categorical variables (participants’ demographics and professional experience) were expressed as numbers and percentages, while continuous variables (age and years of practice) were expressed as means and standard deviations (SD). A one-way analysis of variance (ANOVA) was conducted to determine the factors influencing the pediatricians’ knowledge and attitudes. Multiple regression analysis was performed on the significant factors. Prior to conducting the multiple linear regression analyses, the assumptions of normality, homoscedasticity, and absence of multicollinearity were examined. Statistical significance was set at *p* < 0.05.

### 2.6 Ethical considerations

The study was reviewed and approved by the Hunan Children’s Hospital Research Ethics Committee (reference number: HCHLL-2024-09).

## 3 Results

### 3.1 Sample characteristics

Of the participants, 72.7% were female (*n* = 186). Most were over 29 years of age. More than half (60.9%) of the participants had obtained a master’s degree or higher. More than half of the participants (60.9%) held senior titles. In terms of professional work experience, 69.1% (*n* = 177) had led clinical research projects previously. Almost all (97.7%) pediatricians’ hospitals had ethics committees. More than half (*n* = 171) had attended training related to ethical knowledge. The other general characteristics are presented in Table 1.

**Table 1:** General information and results of univariate analysis of pediatricians’ research ethics knowledge and attitude scores (*x* ± *s*, *n* = 256).

Variant	*n*	%	Knowledge (raw score)	Attitude (raw score)
Genders				
Male	70	27.3	74.03 ± 11.28	27.66 ± 1.72
Female	186	72.7	69.24 ± 10.87	27.93 ± 1.90
*t*			1.333	0.092
*P*			0.249	0.762
Age (years)				
18–28	24	9.3	61.38 ± 11.01	27.75 ± 2.69
29–38	112	43.2	68.90 ± 10.12	27.94 ± 1.82
39–48	110	42.5	70.47 ± 9.47	27.77 ± 1.75
49–58	10	3.9	72.78 ± 11.94	28.10 ± 0.88
*F*			7.467	0.228
*P*			<0.01**	0.877
Degree				
Specialized and below	46	18.0	66.71 ± 9.67	28.35 ± 1.75
Bachelor’s degree	54	21.1	68.78 ± 11.17	27.62 ± 1.75
Master’s degree	149	58.2	71.37 ± 11.04	28.06 ± 2.15
Ph. D./Post-doctoral	7	2.7	75.24 ± 10.19	28.00 ± 1.83
*F*			5.009	2.107
*P*			<0.01**	0.100
Technical title				
Elementary title	31	12.1	69.58 ± 13.10	28.39 ± 0.99
Intermediate title	59	23.0	70.24 ± 9.99	27.90 ± 2.06
Senior title	156	60.9	71.46 ± 11.23	27.79 ± 1.75
None of the above	10	3.9	61.30 ± 5.91	27.00 ± 3.46
*F*			2.779	1.652
*P*			0.042*	0.178
Years of experience				
0–5	59	23.0	67.73 ± 10.30	27.93 ± 1.97
6–10	60	23.4	71.38 ± 8.79	28.10 ± 1.98
11–15	98	38.3	71.65 ± 12.11	27.47 ± 1.78
16–20	11	4.3	73.82 ± 14.54	28.82 ± 0.98
>20	28	10.9	69.57 ± 12.13	28.14 ± 1.63
*F*			1.567	2.307
*P*			0.184	0.059
Number of participation in clinical research projects				
0	9	3.5	71.44 ± 19.31	27.69 ± 1.80
1–3	155	60.5	70.69 ± 11.37	27.72 ± 1.89
4–10	78	30.5	69.76 ± 10.13	28.44 ± 0.88
>10	14	5.5	72.86 ± 8.23	29.93 ± 0.27
*F*			0.355	7.115
*P*			0.785	<0.01**
Number of clinical research projects chaired				
0	79	30.9	69.34 ± 11.33	27.34 ± 1.85
1–3	148	57.8	70.43 ± 11.42	28.51 ± 1.77
4–10	29	11.3	74.45 ± 8.58	28.69 ± 1.07
*F*			2.260	14.900
*P*			0.106	<0.01**
Whether there is an ethics committee in our hospital				
Yes	250	97.7	70.99 ± 10.92	27.83 ± 1.87
No	6	2.3	52.00 ± 0.00	29.00 ± 0.00
*t*			4.253	1.535
*P*			<0.01**	<0.01**
Have you participated in training related to ethical knowledge				
Yes	171		77.50 ± 9.09	28.12 ± 1.38
No	77		66.42 ± 9.71	27.36 ± 2.59
Not sure	8		72.09 ± 11.36	27.00 ± 1.07
*F*			8.976	5.451
*P*			<0.01**	<0.01**

* *P* < 0.05, ** *P* < 0.01.

### 3.2 Knowledge and attitude scores of pediatricians on research ethics

In this study, 256 pediatricians had a total score of 70.55 and 27.86 for research ethics knowledge and attitudes, with standard scores of 70.55 and 92.87, respectively, with moderate-to-high knowledge scores and high attitudinal scores (shown in Tables 2, 3).

**Table 2:** Knowledge and attitude scores on research ethics among pediatricians (*n* = 256).

Projects	Raw score ( $\bar{x}+s$ )	Standardized % score ( $\bar{x}+s$ )
Total knowledge score	70.55 ± 11.17	70.55
Total attitude score	27.86 ± 1.85	92.87

**Table 3:** Three low-scoring entries on the dimension of research ethics knowledge among pediatricians.

Projects	Minimum value	Maximum value	Standardized % score
Basic principles of medical ethics	0	10	3.75 ± 0.85
Scenario judgment 1	0	7	3.50 ± 0.51
Scenario judgment 2	0	7	1.69 ± 0.34

### 3.3 One-way analysis of pediatricians’ research ethics knowledge and attitude scores

There were statistically significant differences in the scores of scientific research ethics knowledge and attitude based on whether there is an ethics committee in our hospital, and whether we have participated in training (*p* < 0.01), as shown in Table 1.

### 3.4 Multiple linear regression analysis of pediatricians’ research ethics knowledge and attitude scores

Knowledge of research ethics and attitude scores were used as dependent variables, and the independent variables that were statistically significant in the univariate analysis were analyzed using multiple linear regression (*α* in = 0.05, α out = 0.10). After multiple linear regression analysis, it was found that degree, technical title, whether there is an ethics committee in our hospital, and whether the participant had participated in the training related to ethics knowledge were the influencing factors of knowledge of research ethics; the number of clinical research projects participated in, the number of clinical research projects presided over, and whether the participant had participated in the training related to ethics knowledge were the influencing factors of the attitude toward research ethics, and the regression coefficients were statistically significant (*p* < 0.05), as shown in Table 4.

**Table 4:** Multiple linear regression analysis of pediatricians’ research ethics knowledge and attitudes (*n* = 256).

Dependent variable	Independent variable	b	Sb	b’	*t*	*P*
Knowledge	Constant	77.743	3.747	–	20.746	<0.001
	Age	1.875	1.034	0.120	1.812	0.071
	Degree	1.866	0.795	0.136	2.347	0.020
	Technical title	1.987	0.924	0.134	2.150	0.033
	Whether there is an ethics committee in our hospital (yes = reference group)					
	No	−18.335	4.512	−0.249	−4.064	<0.001
	Do you participate in training related to ethical knowledge (Yes = reference group)					
	No	−5.993	1.462	−0.247	−4.099	<0.001
	Not sure	6.397	3.737	0.100	1.712	0.088
Attitude	Constant	27.868	0.525	–	53.067	<0.001
	Participated in clinical research projects	0.425	0.182	0.148	2.331	0.021
	Number of clinical research projects chaired	0.437	0.192	0.146	2.273	0.024
	Whether there is an ethics committee in our hospital (Yes = reference group)					
	No	0.792	0.760	0.065	1.041	0.299
	Whether they have participated in training related to ethical knowledge (Yes = reference group)					
	No	−0.755	0.253	−0.187	−2.986	0.003
	Not sure	−0.844	0.657	−0.079	1.285	0.200

## 4 Discussion

This study found that pediatricians’ research ethics knowledge scored at a moderate level (70.55), while their attitudes scored relatively high (92.87). According to Rest’s four-component model, moral judgment (knowledge) often relies on systematic learning and mastery of rules, whereas moral motivation (attitude) is more susceptible to environmental support and cultural climate (25). Pediatricians scored higher on research ethics knowledge than clinical researchers (59.63) and nurses (63.08) (19, 25). This finding reflects the special concerns regarding research ethics in pediatric medicine. Compared to other clinicians, pediatric research involves a special group of underage subjects, which often requires following more stringent ethical review standards (26–28). The results of this study show a relative advantage when compared with previous studies, where Indian scholars reported a mean research ethics score of 65.2 for clinicians (29). However, due to differences in measurement tools, sample characteristics, and cultural contexts. Direct comparisons cannot be made. The reason for this analysis may be the unique ethical sensitivity of the pediatric field, where pediatricians often need to consider the dual interests of legal guardians and minors simultaneously when it comes to informed consent and risk assessment (30, 31). This complex decision-making scenario objectively requires a more comprehensive ethical knowledge base (32). Research ethics knowledge is a fundamental driver of ethical and rigorous pediatric research. It safeguards the scientific integrity and societal credibility of research outputs, reducing the risk of ethical controversies that can hinder the translation of pediatric research findings into clinical practice. Therefore, improving this knowledge base is not just an educational goal but a prerequisite for advancing the entire ecosystem of child health research. Further analysis showed that the basic principles of medical ethics and the two situational judgment questions based on ethical controversies that often arise in the research process had the lowest scores. This indicates that pediatricians have a better grasp of basic theoretical knowledge in the research ethics section but weaker practical knowledge of complex scenarios in the research process. This may be related to the limited number of research projects hosted or participated in or the more general training on research ethics conducted by the hospital, which was not tailored to the specificity of pediatric research projects (33). Therefore, we need to encourage pediatricians to conduct scientific research and strengthen pediatric-targeted research ethics training. During training, they should be given more opportunities to discuss ethical issues that they may encounter in practice and to conduct relevant studies (34).

The mean score of the attitude dimension in this study was high at 92.87, which was significantly higher than the attitude score of clinical researchers (79.55) (25). This is contrary to the results of a study by Chinese scholar Jieming Wu (31). However, this is consistent with the findings of Ghanaian and German scholars (32, 35). To further regulate research ethics, China’s National Health Commission promulgated the Measures for Ethical Review of Biomedical Research Involving Human Beings (hereinafter referred to as the Measures) in 2023 (36), which put forward the principle of special protection and required that special protection be given to subjects from special populations, such as children. After the introduction of the Measures, the ethical review of research project applications and journal articles has become progressively stricter, and researchers’ ethical awareness has continued to improve. The introduction of national policies, such as the 2023 Measures, may have contributed to an increased awareness of research ethics among pediatricians. However, other institutional factors, such as the stringent review processes of hospital ethics committees and researchers’ personal experiences in manuscript submission, likely also play a significant role in shaping their attitudes. In this study, we found that both knowledge and attitude toward research ethics among pediatricians were related to whether they had participated in training related to ethical knowledge. Pediatricians who participated in ethics training had higher scores for their knowledge and attitudes toward research ethics, consistent with the results of many studies (37, 38). The quality of scientific research cannot be guaranteed without researchers’ ethical awareness, and ethical training is indispensable for improving researchers’ ethical awareness (39). The more often one participates in training, the more innovative and comprehensive ethical knowledge one acquires, and the more positive ethical attitude one has. Translating this finding into practice, training programs for pediatric departments should be designed to address the specific ethical vulnerabilities in child health research, such as complex informed consent processes and risk–benefit assessments for minors. Incorporating interactive case discussions based on real pediatric research scenarios could be particularly effective in bridging the gap between theoretical knowledge (which was moderate) and practical ethical judgment (which scored lower). It is worth noting that factors such as degree and technical titles are related to the knowledge score, but not to the attitude score. Pediatricians with higher degrees had higher knowledge scores, consistent with previous results (40). The study, 60.9% of the participants had a master’s degree or above, with more people at higher education levels. High-degree education at the master’s level and above usually systematically teaches normative knowledge of research ethics and reinforces memorization through assessment. Moreover, high-degree pediatricians often take on more research tasks, and their promotion and access to resources depend on the output of their thesis. Therefore, knowledge of research ethics is more solid. However, there was a negative correlation between the degree and attitude scores. Ethical attitude requires long-term practical reflection and situational training, while the traditional education mode often lacks the in-depth cultivation of ethical sensitivity. Therefore, the score of ethical attitudes is not correlated with the degree level. The higher the technical title, the higher the knowledge scores, which is consistent with the results of most international scholarly studies (41, 42). The policy requires physicians to regularly complete continuing education hours, and credits can be converted for academic exchanges and research achievements (43). Therefore, high-level pediatricians are more actively involved in scientific research activities and training because of the demand for career development, thus enhancing their knowledge base.

It is important to note that the subgroup of pediatricians reporting no ethics committee in their hospital was very small (*n* = 6). The primary insight remains that the nearly ubiquitous presence of ethics committees in these tertiary hospitals is associated with a higher baseline level of ethical knowledge. Another point of finding was that the number of hosting and participating in clinical research projects was related to pediatricians’ attitude scores, but not to knowledge scores. Pediatricians who hosted and participated in more clinical research projects had higher attitude scores. Physicians who lead more clinical research projects typically have extensive research and practice experiences that may reinforce their recognition of the value of medical research and thus demonstrate more positive attitudes (44, 45). Clinical research requires teamwork, ethical review, and patient communication. These processes subconsciously enhance the professionalism of pediatricians and have a greater ethical awareness among pediatricians who lead and participate in clinical research projects. In addition, when they hosted and participated in clinical research, they must have really experienced the ethical review in the whole process from project declaration, project implementation, mid-term evaluation and project completion. Therefore, ethical thinking and awareness were also strengthened during the review process, which in turn led to a better attitude toward research ethics.

## 5 Limitations

There are limitations need to be acknowledged. (1) the participants were pediatricians in a province’s tertiary-level hospital, it is possible that this sample was not representative and (2) the univariate analysis involved multiple comparisons across variables, which increases the risk of Type I error even with *p* < 0.05; therefore, its results primarily guided variable selection for the multivariate regression model. Therefore, the differences in research ethics knowledge and attitudes among pediatricians in different provinces, regions, and classes of hospitals in China need to be studied. It is recommended that a larger sample, multicenter comparative survey study of different categories of physicians in each province be conducted in the future. Additionally, the level of pediatricians’ knowledge and attitudes can be further explored, and facilitating and inhibiting factors affecting knowledge and attitudes can be analyzed. This will provide a reference for personalized and precise training on research ethics for pediatricians, and develop appropriate countermeasures and management plans for improving the level of ethical knowledge and attitudes among pediatricians.

## 6 Conclusion

Although these pediatricians had positive attitudes toward research ethics, their knowledge of research ethics was inadequate. This was especially true for the basic principles of medical ethics and two “situational judgment questions” that possessed ethical controversies.” In addition, the degree, technical title, and the existence of an ethics committee in the institution determined the pediatricians’ knowledge of research ethics. Leading and participating in clinical research projects was associated with attitude levels toward research ethics. Participation in ethics-related training simultaneously influenced both knowledge and attitude levels in research ethics. Therefore, hospital research management should establish a comprehensive review process that takes into account local conditions and the special characteristics of children. The lowest scoring items and the factors affecting them should also be taken into account when setting up training courses on research ethics.

## Data Availability

The original contributions presented in the study are included in the article/Supplementary material, further inquiries can be directed to the corresponding authors.

## Footnotes

1
www.wjx.cn
