# Supplementary material for: Current status and factors influencing research ethics knowledge and attitudes among pediatricians: a cross-sectional survey
Source: Front Med (Lausanne). 2026 Jan 15;12:1697458. doi: 10.3389/fmed.2025.1697458 (PMC12852005; doi:10.3389/fmed.2025.1697458)
Supplement: Supplementary file 1 [file Table_1.docx]

**Questionnaire on Ethical Cognition and Attitude of research ethics among pediatricians**

Greetings! Thank you for taking the time to participate in this questionnaire, the purpose of our survey was to understand pediatricians cognition and attitudes towards ethical issues involved in clinical research. This questionnaire was filled out anonymously and the results of the survey are for academic research purposes only. Please be assured that we promise to keep your private information strictly confidential. We would like to express our sincere gratitude for your active participation and cooperation!

. **Basic Information**

1. Genders[Single choice]*

○Male

○Female

2. Age[Single choice] *

○18-28 years old

○29-38 years old

○39-48 years old

○49-58 years old

○58 years old or older

3. Degree[Single choice]*

○Specialist and below

○Bachelor's Degree

○Master

○PhD/Postdoctoral

4. Technical title [Single choice]*

○Junior

○Intermediate

○Senior

5. Years of experience[Single choice]*

○0-5 years

○6-10 years

○11-15 years

○16-20 years

○＞ 20 years

6.Number of participation in clinical research projects (items)[Single choice]*

○0 items

○1-3 items

○4-10 items

○＞10 items

7. Number of clinical research projects chaired (items)[Single choice]*

○0 items

○1-3 items

○4-10 items

○＞10 items

8. Whether there is an ethics committee in our hospital[Single choice]*

○Yes

○No

9.Have you participated in training related to ethical knowledge [Single choice] *

○Yes

○No

**Pediatricians cognition of clinical research ethics**

1. The following is a correct statement of "ethics": [Single choice] *A(7)

○The principles and guidelines that should be followed when dealing with human beings and society.

○The way to get along with others

○ Mutual respect between personalities

○Moral restraints on people

○Unknown

2. Basic Principles of Medical Ethics [Multiple choice] *ABCD (10)

□No harm

□Favorable

□Respect

□Justice

□Unknown

3. The Declaration of Helsinki of the World Medical Assembly refers to: [Single choice] *B (7)

○ A normative document for clinical research

○Ethical principles and limitations for biomedical research using human beings as test subjects

○International Principles of Medical Ethics

○Methods for the management of quality control of clinical research

○Unknown

4. During what period of time do ethics committees review clinical research? [Single choice] *(7)A

○Before the declaration of clinical research

○After a clinical research project has been declared

○Before the implementation of clinical research

○During clinical research

○Not specified, not clear

5. The contents of the ethics committee review are: [Single choice] *(7)E

○Project/subject bids

○Research protocol

○Version of informed consent

○Application for ethical review

○All of the above

6. The central role of ethical review for clinical research is to: [Single choice] *(7) B

○ Constraining researchers

○ To protect the rights and interests of research subjects

○to promote sound science

○Reviewing research protocols

○Assessment of the declaration of clinical research

7. Do all research subjects need to sign an informed consent form before clinical research? [Single choice ] *(7)A

○Must sign

○Not necessary

○It depends

○It is not clear

8. Informed consent involves: [Multiple choice] *(10) ABCDE

□ Background of the study

□Purpose of the study

□Methodology of the study

□Potential risks

□Patient benefits

9. Requirements for research subjects to sign the informed consent form: [Multiple choice] *(10) ACD

□ Signed by the subject himself/herself

□A friend or relative signs for the participant

□An immediate family member signs for the participant

□Legal guardian signs

□ Someone's signature is sufficient

□I am not sure

10. In your opinion, the most important part of a clinical study is: [Single choice] *(7)A

○Safety of research subjects

○Advancement of life science

○Successful completion of the project/subject

○Data collection

○Results of the study

11. The Chinese name of GCP is: [Single choice] *(7)B

○Multi-center trial standard

○Criteria for Quality Management of Drug Clinical Trials

○Clinical Research Practice Techniques

○Study quality control

○Don't know

12. According to the diagnostic and treatment guidelines, a child needs to undergo a renal biopsy before treatment to clarify the etiology of the disease. If, due to the need for efficacy control in a clinical study, the study protocol requires that a renal biopsy be performed before and after treatment, do you think that a renal biopsy should be performed after treatment? [Multiple choice question] *C (7)[Single choice]

○ Should, to be practiced according to the established study protocol.

○Should, before and after comparisons are better for assessing treatment efficacy and subsequent treatment regimens.

○No, renal biopsy after treatment is no longer informative for diagnosis and treatment, is overmedication, and is against ethical principles as the child is subjected to secondary injuries.

○Depending on the child's recovery and physical tolerance.

○ It is up to the child/guardian to decide.

○Don't know

13. If two tests, A and B, are routinely required for the review of a child with a certain disease, but four tests, A, B, C, and D, are required in the clinical research protocol to compare the clinical efficacy, do you think two tests, C and D, should be performed? [Multiple choice question] *E (7)[Single choice]

○ Should, to be practiced according to the established study protocol.

○Should, there is no harm to the child if 2 more tests are performed

○Should not, C and D tests are not meaningful for the assessment of the child's recovery

○No, it will increase the financial burden of the child's family.

○It is up to the child/guardian to decide.

○Don't know

**Pediatricians ethical attitudes toward clinical research**

1.Do you think that ethical committee review is required to conduct clinical research? *

A. Strongly agree

B.Quite agree

C.Not sure

D. Basically disagree

E. Strongly disagree

2. Do you think the ethics committee has the right to question or modify the research protocol? *

A.Strongly agree

B.Quite agree

C.Not sure

D.Basically disagree

E. Strongly disagree

3. Do you think that any changes in the clinical research protocol during the study need to be reported to the ethics committee? *

A. Strongly agree

B. Quite agree

C.Not sure

D. Basically disagree

E. Strongly disagree

4. The ethics committee has the right to order suspension/stoppage of the trial in case of adverse events during the clinical study? *

A. Strongly agree

B.Quite agree

C.Not sure

D.Basically disagree

E. Strongly disagree

1. Do you think that ethical review is needed before clinical research:

A.Strongly agree

B.Quite agree

C.Not sure

D.Basically disagree

E.Strongly disagree

1. Do you think GCP certificate is needed to conduct clinical research?

A.Strongly agree

B.Quite agree

C.Not sure

D.Basically disagree

E.Strongly disagree
